# 3D computer modeling of inhibitors targeting the MCF-7 breast cancer cell line

**DOI:** 10.3389/fchem.2024.1384832

**Published:** 2024-05-23

**Authors:** Sara Zarougui, Mohammed Er-Rajy, Abdelmoujoud Faris, Hamada Imtara, Mohamed El fadili, Ashraf Ahmed Qurtam, Fahd A. Nasr, Mohammed Al-Zharani, Menana Elhallaoui

**Affiliations:** ^1^ Laboratory of Engineering, Modelisation and Systems Analysis, Department of Chemical Sciences, Faculty of Sciences Dhar El Mahraz, Sidi Mohamed Ben Abdellah University, Fez, Morocco; ^2^ Faculty of Medicine, Arab American University Palestine, Jenin, Palestine; ^3^ Department of Biology, College of Science, Imam Mohammad Ibn Saud Islamic University (IMSIU), Riyadh, Saudi Arabia

**Keywords:** MM/GBSA, 3D-QSAR, molecular docking, molecular dynamics simulation, MCF-7 cell line

## Abstract

This study focused on developing new inhibitors for the MCF-7 cell line to contribute to our understanding of breast cancer biology and various experimental techniques. 3D QSAR modeling was used to design new tetrahydrobenzo[4, 5]thieno[2, 3-d]pyrimidine derivatives with good characteristics. Two robust 3D-QSAR models were developed, and their predictive capacities were confirmed through high correlations [CoMFA (Q^2^ = 0.62, *R* ^2^ = 0.90) and CoMSIA (Q^2^ = 0.71, *R* ^2^ = 0.88)] via external validations (R^2^_ext_ = 0.90 and R^2^_ext_ = 0.91, respectively). These successful evaluations confirm the potential of the models to provide reliable predictions. Six candidate inhibitors were discovered, and two new inhibitors were developed *in silico* using computational methods. The ADME-Tox properties and pharmacokinetic characteristics of the new derivatives were evaluated carefully. The interactions between the new tetrahydrobenzo[4, 5]thieno[2, 3-d]pyrimidine derivatives and the protein ERα (PDB code: 4XO6) were highlighted by molecular docking. Additionally, MM/GBSA calculations and molecular dynamics simulations provided interesting information on the binding stabilities between the complexes. The pharmaceutical characteristics, interactions with protein, and stabilities of the inhibitors were examined using various methods, including molecular docking and molecular dynamics simulations over 100 ns, binding free energy calculations, and ADME-Tox predictions, and compared with the FDA-approved drug capivasertib. The findings indicate that the inhibitors exhibit significant binding affinities, robust stabilities, and desirable pharmaceutical characteristics. These newly developed compounds, which act as inhibitors to mitigate breast cancer, therefore possess considerable potential as prospective drug candidates.

## Introduction

All living beings, including humans, animals, and various organisms, can be affected by cancer; this disease does not exhibit favoritism and can affect individuals in different ways. Both men and women are vulnerable to cancer, but there are certain types of cancers that are more common in each gender. Prostate, lung, and colon cancers are frequently diagnosed in men, whereas breast, lung, and colon cancers are more prevalent in women. Young individuals are often affected by leukemia, brain tumors, and lymphomas as the most common types of cancer. Sadly, certain types of cancers consistently contribute toward a significant number of deaths, i.e., lung, stomach, liver, and colon cancers, which require urgent attention to reduce fatalities (Zaorsky et al., 2017). There are various cancer treatment options available that can be categorized as either local or systemic. Local treatments include surgery, hyperthermia therapy, and radiation, whereas systemic treatments comprise chemotherapy, hormone therapy, and targeted therapy. In most cases, individuals undergo a combination of treatments, such as surgery accompanied by chemotherapy and/or radiotherapy. Additionally, neoadjuvant chemotherapy, which involves using chemotherapy to reduce the size of a tumor before radiation or surgery, is another approach that is utilized frequently. Our focus here is on chemotherapy involving the use of drugs to address cancerous growths in various parts of the body (Falzone et al., 2018). The central fraction with anticancer properties is mainly composed of the pyrimidine group; additionally, the thieno[2,3-d]pyrimidine ring, which is a bioisostere of quinazoline, has been used as a central structure in the design and synthesis of various compounds with potential antitumor activities (Dai et al., 2005). The thienopyrimidine nucleus has also been utilized as a molecular framework in the development of numerous antimicrobial (El-Sherbeny et al., 1995; Aly et al., 2011), antiviral (El-Sherbeny et al., 1995; Rashad et al., 2010), anti-inflammatory (Alagarsamy et al., 2006), antidiabetic (Deng et al., 2011), antioxidant (Kotaiah et al., 2012), and anxiolytic (Amr et al., 2010) agents. Prior studies in literature have documented that the antitumor activity of the thieno[2,3-day]pyrimidine core can be maintained by fusing it with cycloalkyl lipophilic moieties with five, six, or seven members (Zhang et al., 2020; Ouassaf et al., 2021). This is evident in the case of the 4-substituted-5,6,7,8-tetrahydrobenzo[4,5]thieno[2,3-day]pyrimidine, which exhibits important anticancer activity (Aponte et al., 2010).

**FIGURE 1:**
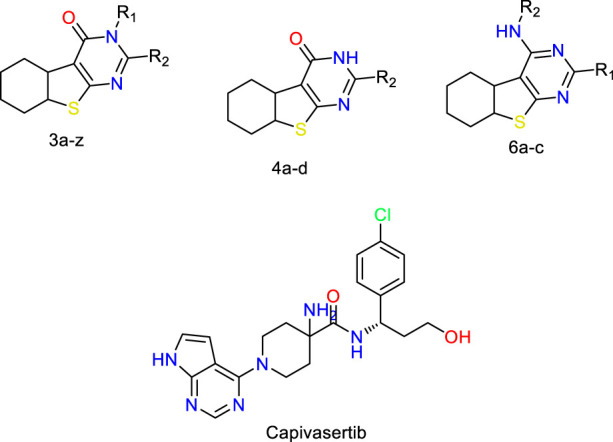
Structures of the 2,3-disubstituted derivatives (3a-z and 4a-d), 2,4-disubstituted derivatives (6a-c), and FDA-approved drug capivasertib.

Breast cancer is now widely recognized as one of the leading types of cancer diagnosed in women across the globe. Malignant tumors in women present significant risks, often resulting in considerable illness and death. Moreover, statistics show that this type of cancer is relatively rare in men (Elbachiri et al., 2017). Breast cancer can be influenced by various risk factors, such as gender, diet, breast density, age, hormonal factors, radiation exposure, obesity, mutations, and family history (Maryam et al., 2017). The US Food and Drug Administration has approved the drug capivasertib (Figure 1) as an AKT inhibitor that works by inhibiting the AKT1 enzyme. By targeting this key signaling pathway, it shuts down the machinery driving cancer cell growth and resilience. In prior studies, MCF-7 cell line cultures were subjected to varying doses of capivasertib to evaluate its effects; as the concentration of the tested agent increased, the cell viability decreased progressively, indicating a dose-dependent inhibitory effect. Higher doses of capivasertib resulted in greater inhibition. Subsequent experiments have revealed that capivasertib treatment reduces cell growth, causes cell cycle arrest, and facilitates cell death. Blocking the AKT1 pathway with capivasertib disrupts the key processes that the MCF-7 cells rely upon to grow and proliferate unchecked like cancer cells (Hopcroft et al., 2023).

In the present work, we use the three-dimensional quantitative structure–activity relationship (3D-QSAR) with the comparative molecular field analysis (CoMFA) and comparative molecular similarity indices analysis (CoMSIA) descriptors to determine the key characteristics for the potential inhibition of MCF-7 cells by tetrahydrobenzo[4,5]thieno[2,3-d]pyrimidine derivatives (Er-rajy et al., 2022; Er-rajy et al., 2023a). In addition, the ADME-Tox (ADMET) studies and pharmacokinetic characteristics were used to predict the pharmaceutical features of the newly designed molecules for aspects crucial to drug effectiveness and pharmacokinetics, including solubility, permeability, and metabolic stability (Alqahtani, 2017). This research also involves investigating the interactions between the protein ERα and ligands using the molecular docking approach. The aim of this investigation is to discover promising compounds that can selectively inhibit the MCF-7 cell line. To achieve this, we conducted molecular dynamics (MD) and molecular mechanics (MM) simulations. Additionally, we utilized MM and generalized Born surface area (MM/GBSA) calculations (Sanachai et al., 2022). Specifically, we performed MD simulations for a duration of 100 ns to evaluate the stabilities of the derived complexes.

## Materials and methods

### Dataset

Twenty-nine derivatives of the novel tetrahydrobenzo[4, 5]thieno[2, 3-d]pyrimidine molecule that showed breast cancer (MCF-7) inhibitory activity were collected from literature (Abbas et al., 2013). The database was randomly split into a training set of 24 derivatives and a test set of five derivatives. The half-maximal inhibitory concentration (IC_50_) values, which measure the abilities of the derivatives to inhibit breast cancer activity, were converted to negative logarithms (pIC_50_ = -log (IC_50_)) for use as the dependent variables during modeling. The investigated structures and their corresponding pIC_50_ values are shown in Table 1. In addition, all molecular structures were analyzed with Powell’s and Gasteiger–Huckel partial atomic charge using the Tripos force field in SYBYL-X.2.1 software (Certara, 2017).

**TABLE 1:** Structures and anti-MCF-7 activities of the molecules studied.

N°	Structure	pIC_50_	N°	Structure	pIC_50_
3a	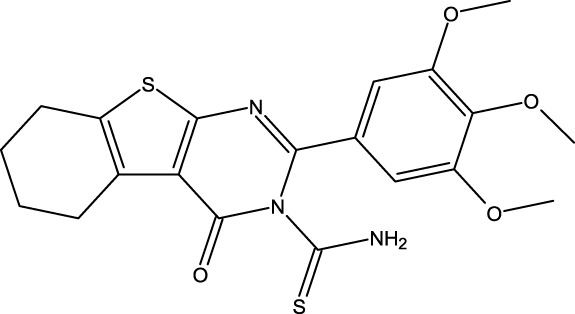	4.474	3q	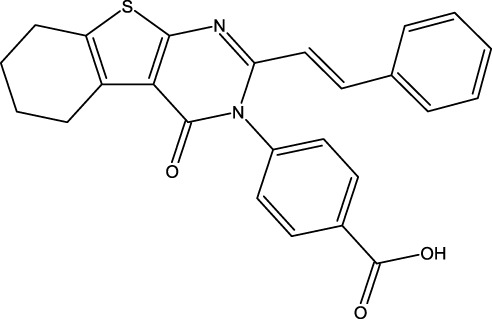	4.712
3b	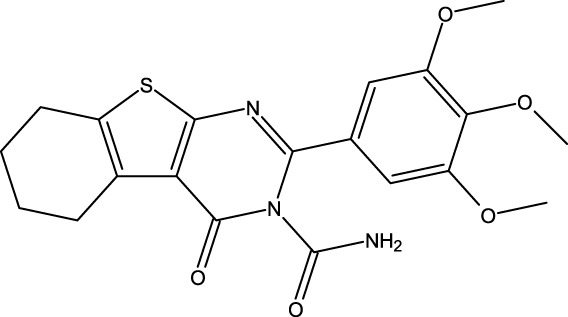	4.962	3r	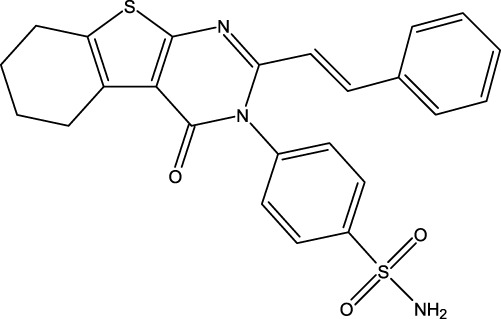	6.698
3c	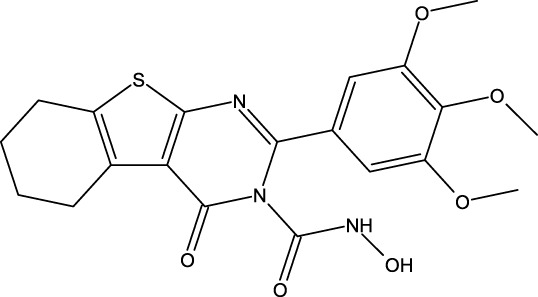	4.512	3s	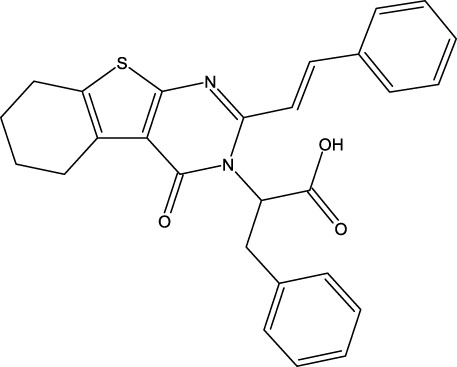	5.180
3e	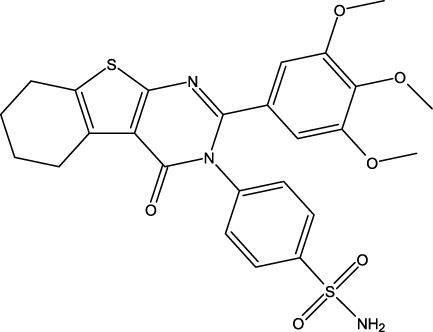	4.707	3u	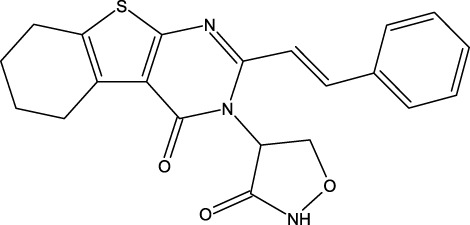	4.809
3f	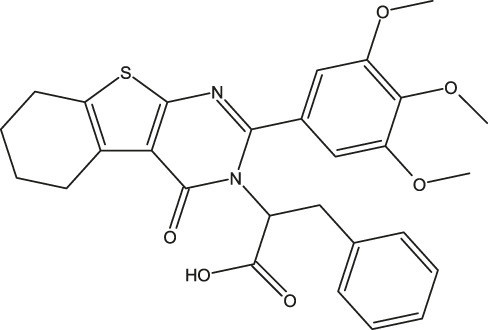	4.583	3v	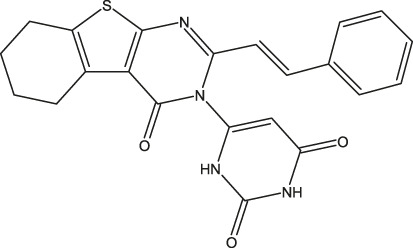	5.327
3h	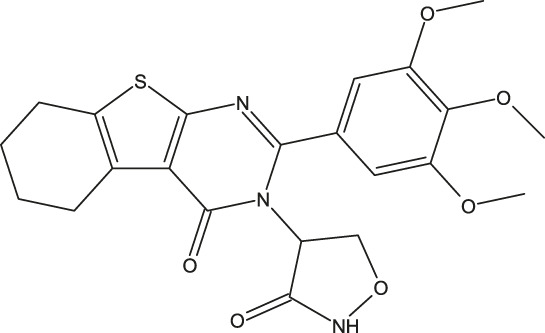	4.600	3w	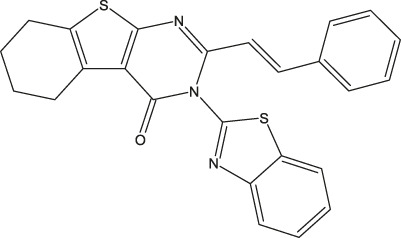	5.721
3i	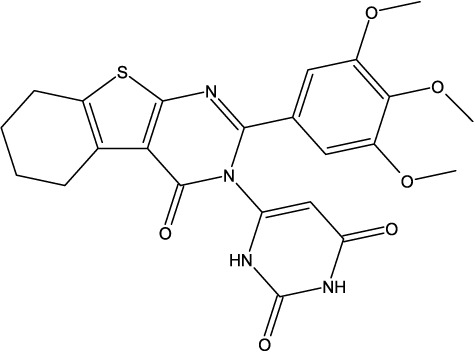	4.395	3x	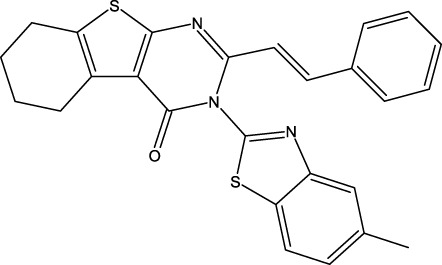	5.142
3j	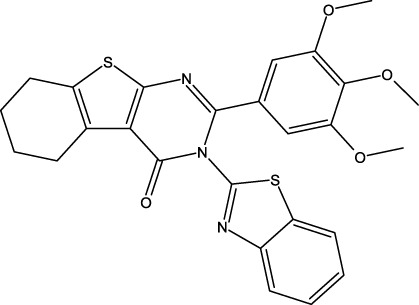	5.236	3y	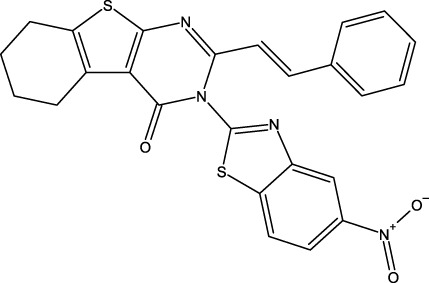	6.221
3k	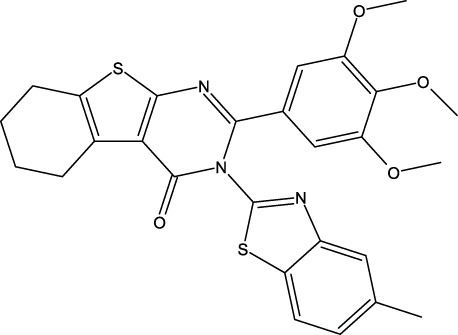	4.467	3z	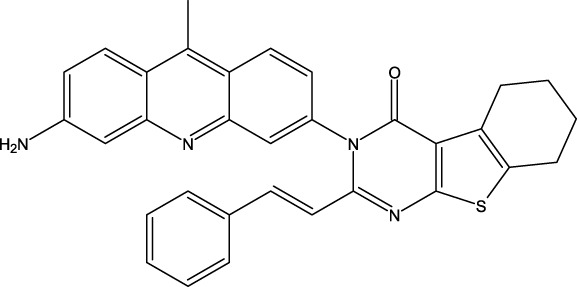	7
3L	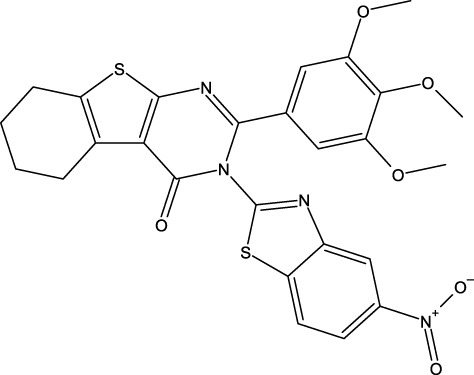	4.924	4b	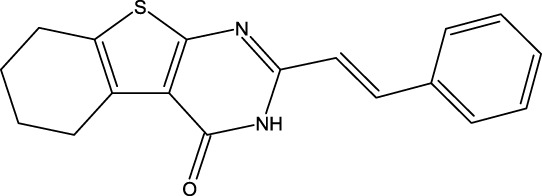	5.060
3m	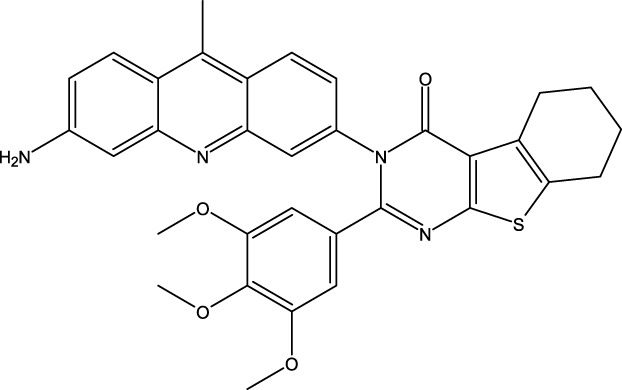	6.301	4c	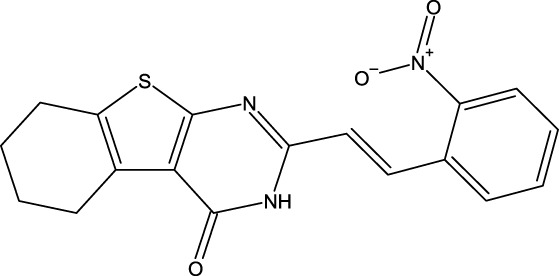	5.376
3n	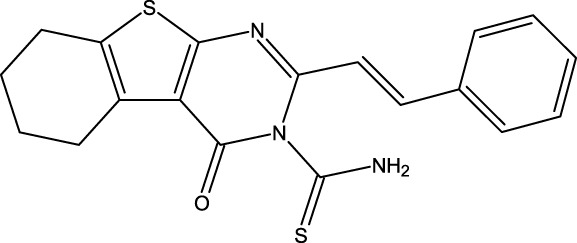	5.408	4d	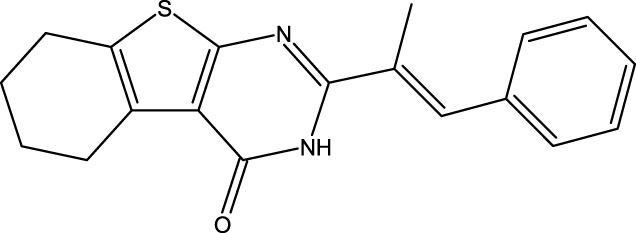	5.096
3o	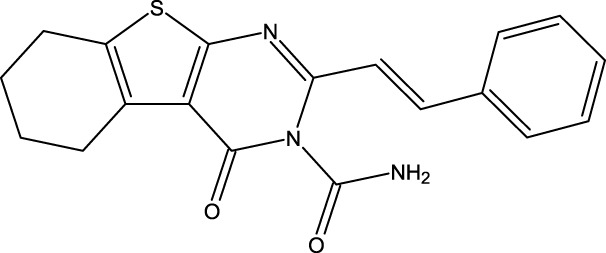	5.327	6a	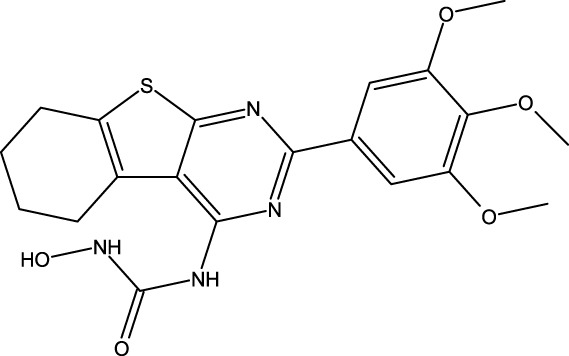	5.214
3p	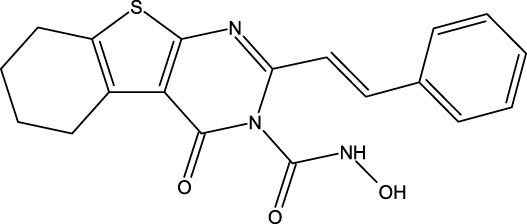	5.055	6b	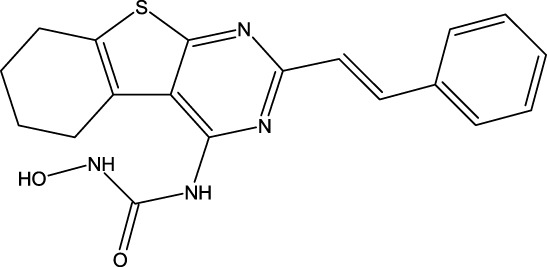	5.619
6c	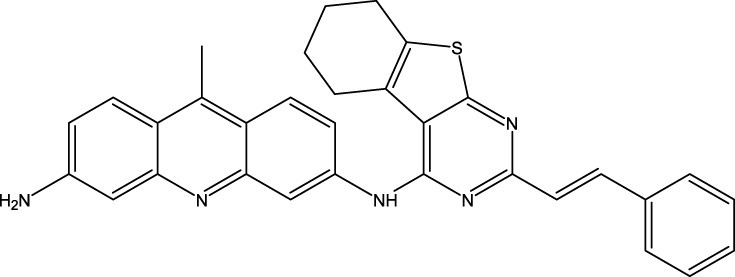	6.522			

### Molecular alignment

In the 3D-QSAR analysis, proper alignment of the molecules is of utmost importance. Molecular alignment plays a critical role in CoMFA and CoMSIA, and it has been widely recognized as a significant factor in these methods, demonstrating great sensitivity (Zarougui et al., 2023). As the first step in building the model, we selected the most potent compound (3z) with a pIC_50_ value of 7 as the template structure. Subsequently, all other molecules were aligned to this template using the distill module in SYBYL 2.1 software by taking into consideration their structural similarities.

### 3D-QSAR modeling

To determine the key structural properties that affected the biological activities of the compounds under investigation, 3D-QSAR models were created using the CoMFA and CoMSIA techniques (Aparoy et al., 2011; Tong et al., 2021). These techniques enabled examination of various molecular characteristics, such as electrostatic, steric, hydrogen-bond donor, hydrophobic, and hydrogen-bond acceptor, and their potential effects on biological activity (Ouassaf et al., 2021). In this research, the quantitative relationships between the 3D field descriptors and MCF-7 breast cancer cell line activities (pIC_50_) were studied through the application of the partial least-squares (PLS) algorithm within the Tripos force field. Specifically, the Tripos force field utilized a reference input lattice defined at 2 Å in all Cartesian directions, with the sp^3^ hybrid carbon atoms serving as the source for the steric and electrostatic energy calculations, and the default cutoff energy set to 30 kcal/mol (Goodhue et al., 2006). In addition, appropriate QSAR models representing the structure–activity relationships of the tested compounds were chosen based on different statistical criteria, notably the coefficient of determination (*R* ^2^), coefficient of determination by cross-validation (LOO-CV Q^2^), standard error of estimate (SEE), and coefficient of determination calculated for all tests (R^2^_pred_) (Golbraikh and Tropsha, 2000; Golbraikh and Tropsha, 2002).

### Molecular docking

Our primary aim in the molecular docking study was to employ computational techniques to forecast the structures of the complexes formed between ligands and their receptors. This process entailed two main phases. First, we sampled various conformations of the ligand within the receptor’s active site, followed by assessments of these conformations using a scoring function. For our investigation, we utilized AutoDock Tools 1.5.6 (My Biosoftware, 2019) and assigned Kollman charges to the protein (3.372) and Gasteiger charges to the 3M (−1) and 5M (−1) molecules (Kumari and Dalal, 2022), while incorporating polar hydrogen atoms. This enabled the removal of water molecules and facilitated visualization of the ligand–protein interactions using Discovery studio (Dassault Systèmes, 2024). Molecular docking was implemented using the cocrystalline structure of the ERα protein (PDB code: 4XO6) as the model. Our focus was specifically on the newly designed compounds with high biological activities (pIC_50_), in addition to drug similarity assessments and ADMET studies, for the molecular docking simulations with the MCF-7 cell line. The 3D-grid defined using AUTOGRID algorithms was crucial for determining the strengths of attraction between the ligands and receptors. The grid center was located at (x = 12.444, y = −0.171, z = 2.943), with default dimensions of (x = 60, y = 60, z = 60) and grid point spacing of 0.375 Å. Finally, after preparing both the ligands and receptors, we conducted molecular docking on the two newly designed molecules, M3 and M5, using AutoDock Tools (My Biosoftware, 2019).

### MD simulation

The newly created compounds that exhibited enhanced binding affinities with ERα were subjected to all-atom MD simulations using the Groningen Machine for Chemical Simulation (GROMACS 2021) software (Van Der Spoel et al., 2005; Abraham et al., 2015). Before implementing the MD simulations, the CHARMM-GUI web server (Jo et al., 2008) was used to generate the initial input parameters by applying the CHARMM36 force (Ziada et al., 2022). The simulations were carried out at pH 7. Before moving to the generating phase, each complex was solved in a rectangular grid encircled by TIP3P water molecules and complemented with the necessary counter ions (Na^+^, Cl^–^) to maintain a salt concentration of 0.15 M, as determined by Monte Carlo ion shifting. Energy minimization was performed for each system using the steepest downslope algorithm with a top of 50,000 steps and top force of 10.0 kJ/mol. The temperature and atmospheric pressure were set to 310 K and 1.01325 bar, respectively. Canonical (NVT) and isothermal–isobaric (NPT) assemblies were used to equilibrate each system, and the MD simulations were performed for a duration of 200 ns. To evaluate the structural stabilities of the newly designed molecules, several parameters like the root mean-squared deviation (RMSD), solvent-accessible surface area (SASA), radius of gyration (RoG), and root mean-squared flexibility (RMSF) were tested for the dynamic path results (Kumari et al., 2014; Faris et al., 2023).

### Free binding energy (MM/GBSA)

The MM/GBSA technique is used to quantify the amount of energy released or absorbed when a ligand binds to a protein, which is known as the free binding energy of a complex (Zhang et al., 2017). We used AMBER 14 software (Case et al., 2023) to determine the binding free energy (Aqvist, 1990; Zhang et al., 2017) using the following equation: ΔG_binding_ = G_complex_ – (G_receptor_ + G_ligand_); here, G_receptor_ is the free energy of the open receptor and G_ligand_ is the ligand’s boundaryless free energy that can be described using thermodynamics. The binding free energy (ΔG_binding_) is also given by the equation ΔG_binding_ = ΔH – TΔS, where ΔH represents the affinity enthalpy and ΔS is the change in entropy after binding (Wang et al., 2019; Valdés-Tresanco et al., 2021). The calculations were performed using the last 200 images by applying the following equations:

$$\Delta G_{\text{bind}}=G_{\text{complex}}\;-\;G_{\text{protein}}\;-\;G_{\text{ligand}}$$ (1)

$$\Delta G_{\text{bind}}=\Delta G_{\text{gas}}+\Delta G_{\text{sol}}\;–\;T\Delta S$$ (2)

$$\Delta G_{\text{gas}}=\text{Bond}+\text{Angle}+\text{Dihed}+E_{\text{EL}}+\text{VdW}$$ (3)

$$\Delta G_{\text{sol}}=\Delta E_{\text{GB}}+\Delta E_{\text{SURF}}$$ (4)

Here, ΔG_bind_ represents the total binding energy change of the system, as given by Eq. (1), and is determined using Eq. (2); TΔS denotes the transformational entropy change resulting from ligand binding at a certain temperature; ΔG_gas_ is the combined contributions from the bond, angle, dihedral, and van der Waals energies as well as the electrostatic component of the internal energy (E_EL_), as explained in Eq. (3). The internal energy is linked to fluctuations and rotations in the torsion angles of the individual bonds. The solvation energy (ΔG_sol_) is composed of the polar property of solvation energy (ΔE_GB_) and non-polar property of solvation energy (ΔE_SURF_), as shown in Eq. (4).

### ADMET prediction

Following the stability studies conducted on the M3 and M5 molecules, their pharmacokinetic and pharmacodynamic properties were examined. Here, the first step involved confirming the feasibility of synthesizing these compounds, while the second step involved investigating the pharmaceutical properties of each compound. To evaluate the synthetic accessibility and pharmaceutical characteristics of the proposed compounds, the pkCSM (Pires et al., 2015) and SwissADME servers (Daina et al., 2017) were utilized.

## Results and discussion

### Rigid molecular alignment method

**FIGURE 2:**
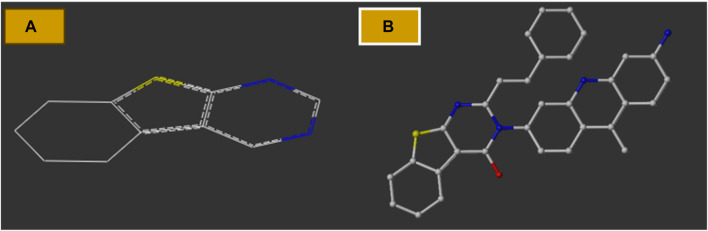
**(A)** Common structure; **(B)** two-dimensional template skeleton.

**FIGURE 3:**
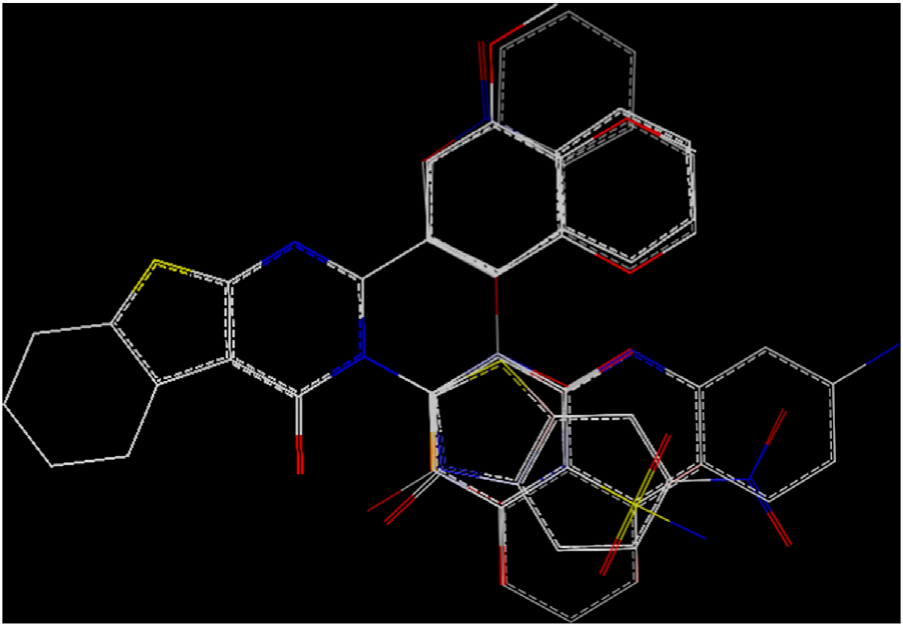
Molecular alignment.

Structural alignment of a molecule is key to the predictive precision of a 3D-QSAR model and accuracy of a contour model (Er-rajy et al., 2023c). The tetrahydrobenzo[4,5]thieno[2,4-day]pyrimidine and tetrahydrobenzo[4,5]thieno[2,3-day]pyrimidine molecules were first optimized and then aligned on the basis of their similarities with the template skeleton (3z) displayed in Figure 2A. Figure 2B demonstrates that all the examined molecular structures are appropriately aligned with the template structure. The shared core of all the overlaid molecules is depicted in Figure 3.

### PLS analysis

PLS analysis was conducted to investigate the correlation between pIC_50,_ and the 3D field descriptors obtained from the CoMFA and CoMSIA techniques. There are significant correlations between the inhibitory activities of the MCF-7 cells (measured as pIC_50_) and two descriptors: steric and electrostatic. The strong predictive abilities of our developed models are evident from the high R^2^_pred_ values [R^2^_pred_ (CoMFA) = 0.90 and R^2^_pred_ (CoMSIA) = 0.91] obtained from external validations (Table 2). This predictive power is further supported by the very low residuals between the observed pIC_50_ values *in vitro* and predicted pIC_50_ values *in silico* (Table 3).

**TABLE 2:** CoMFA and CoMSIA statistics results based on PLS regression.

	Q^2^	*R* ^2^	R^2^_pred_	N	SEE	Fractions	
Metrics						Steric	Electrostatic
CoMFA	0.62	0.90	0.90	5	0.24	0.56	0.43
CoMSIA	0.71	0.88	0.91	9	0.27	0.55	0.44

Q^2^, cross-validation correlation factor; N, optimum value of components determined by cross-validation (LOO-CV); SEE, standard error estimation; *R* ^2^, standard coefficient of determination; R^2^_pred_, coefficient of determination based on the external test; fractions, participation of steric and electrostatic interactions.

**TABLE 3:** pIC_50_ values observed and predicted by the 3D-QSAR (CoMFA and CoMSIA) models.

Compound	MCF-7 pIC_50_	CoMFA		CoMSIA	
		Predicted (pIC_50_)	Residual	Predicted (pIC_50_)	Residual
3a	4.47	4.42	0.05	4.44	0.03
3b^a^	4.92	4.43	0.49	4.60	0.32
3c^a^	4.51	4.41	0.09	4.69	−0.18
3e	4.70	4.50	0.19	4.69	0.016
3f	4.58	4.41	0.16	4.54	0.042
3h^a^	4.60	4.25	0.34	4.88	−0.28
3i	4.39	4.60	−0.20	4.47	−0.08
3j	5.23	4.82	0.41	5.10	0.13
3k	4.46	4.75	−0.28	4.45	0.01
3L	4.92	5.36	−0.43	5.15	−0.23
3m	6.30	6.38	−0.08	6.30	−0.004
3n^a^	5.40	5.11	0.29	5.31	0.096
3o	5.32	5.13	0.19	5.38	−0.054
3p	5.05	5.10	−0.05	4.99	0.058
3q	4.71	5.14	−0.43	4.73	−0.02
3r^a^	6.69	5.06	1.63	6.68	0.01
3s	5.18	5.09	0.08	5.17	0.004
3u	4.80	4.90	−0.09	4.83	−0.02
3v	5.32	5.32	0.005	5.26	0.06
3w	5.72	5.49	0.22	5.90	−0.18
3x	5.14	5.27	−0.13	5.17	−0.03
3y	6.22	6.06	0.15	5.99	0.22
3z	7	6.78	0.21	6.97	0.02
4b	5.06	5.28	−0.22	5.09	−0.03
4c	5.37	5.21	0.15	5.35	0.02
4d	5.09	5.07	0.02	5.05	0.04
6a	5.21	5.06	0.14	5.12	0.08
6b	5.61	5.60	0.01	5.70	−0.08
6c	6.52	6.62	−0.10	6.53	−0.01

^a^ Test set.

Based on the information in Table 2, it is observed that the electrostatic and steric forces that contribute 37% and 63%, respectively, to the overall variance represent the CoMFA descriptor. The Q^2^ value obtained by adjusting the leave-one out (LOO) step in the PLS analysis is 0.62. Additionally, *R* ^2^ is reported to be 0.90, with the number of components being five. This high *R* ^2^ value also has strong external validation for the test set, with an R^2^_Test_ of 0.90. The CoMSIA descriptor is characterized by two fields, namely, the electrostatic and steric fields, which respectively contribute 44.6% and 55.4%. The Q^2^ value adjusted through LOO-CV in the PLS method is 0.71. Additionally, *R* ^2^ is evaluated to be 0.883, indicating a lower level of correlation. However, when considering the nine components, the high *R* ^2^ value suggests robust external validation for the test set, with an R^2^_Test_ of 0.91.

### Contour maps of CoMFA and CoMSIA

**FIGURE 4:**
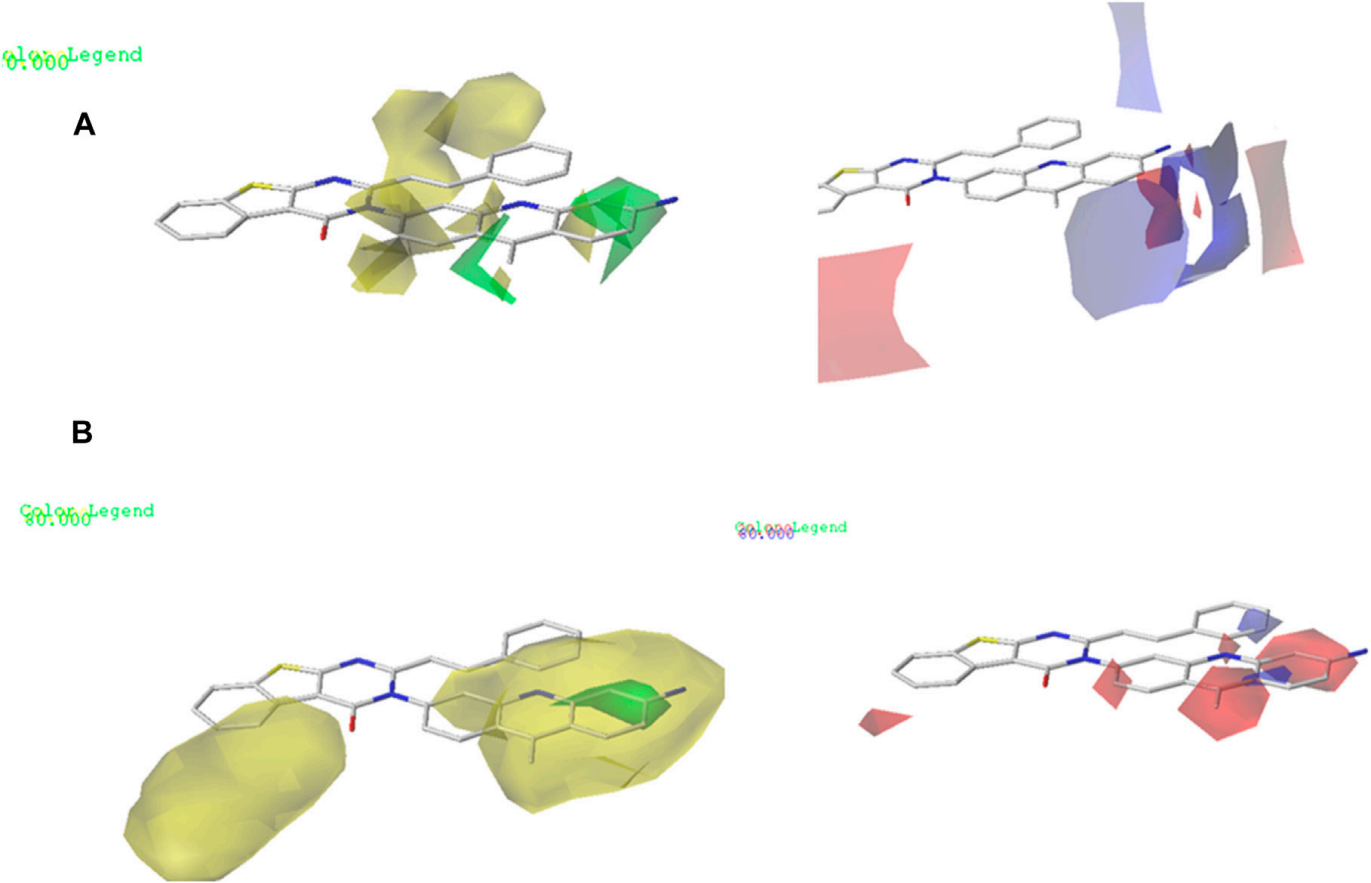
**(A)** CoMFA contour maps: electrostatic area interactions (blue = promising/red = unpromising) and steric area interactions (green = promising/yellow = unpromising). **(B)** CoMSIA contour maps: electrostatic area interactions (blue = promising/red = unpromising) and steric area interactions (green = promising/yellow = unpromising).

**FIGURE 5:**
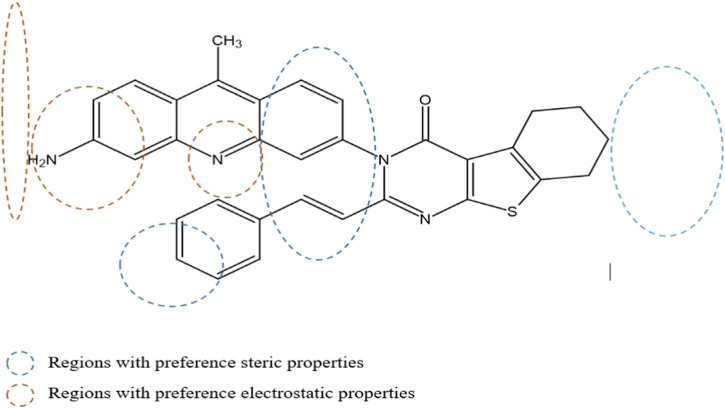
Mapping regions that are favorable for biological activity against the MCF-7 cell line.

The contour maps in the QSAR models represent the relationships between the structures and activities of the series of chemicals. In this study, we analyzed the contour maps of the developed models to determine the factors influencing their anticancer activities (Er-rajy et al., 2023b). By conducting a three-dimensional analysis of these visualizations, we identified the regions in the template molecule that require modifications for designing new molecules with enhanced biological activities compared to the investigated derivatives. Figure 4 and Figure 5 depict the 3D contour maps of the template molecule structure generated using the CoMFA and CoMSIA models, respectively. The steric zone can be visualized using two colors; the presence of the green edges (which contribute to 80% of the zone) indicates regions where expansive clusters enhance the biological activity. On the other hand, the yellow areas (contribution amounting to 20%) represent regions where the voluminous clusters diminish the biological activity. The electrostatic zone can be visualized using two colors as well; the electrostatic field is deemed positive where the blue contours prevail (making up 80% of the zone), whereas the red edges (making up 20% of the zone) indicate the promotion of negative electrostatics. The CoMFA steric contours reveal a prominent green area surrounding the substituent in the Meta Position 2, suggesting that a bulky group is required for R substitution at the Meta position number 2 for the tetrahydrobenzo[4,5]thieno[2,3-day]pyrimidine derivative in this region to enhance the biological activity. The CoMFA electrostatic contours suggest that the introduction of strongly electropositive groups or atoms in this position may improve activity. In the CoMSIA electrostatic zone, we observed extensive yellow-colored contours surrounding the 9-methylacrinidin-3-amine substitution at the Meta position for the tetrahydrobenzo[4,5]thieno[2,3-day]pyrimidine derivatives. In addition, we noted red borders in the vicinities of the 9-methylacridin-3-amine substitutions in the Meta position number 3 of the compounds. This shows that the substitution of highly electronegative compounds or atoms can increase the activity of the tetrahydrobenzo[4,5]thieno[2,3-day]pyrimidine derivatives.

### Design of new derivatives as inhibitors of MCF-7 cells

The ADMET analysis is crucial in the process of developing and discovering new drug compounds as it enables assessment of their pharmacological effects and potential risks more effectively. In our study, we specifically focused on investigating the effects of (3z), the most active compound, as well as the two newly designed compounds (M3 and M5), as presented in Table 6. Additionally, we examined the ADMET properties of capivasertib, an FDA-approved drug, to compare its results with those of the newly developed molecules. The ADMET findings are provided in Table 4. Here, 3z displayed a logS value of −7.84, indicating significantly reduced solubility; the compound also exhibited an exceedingly high LogP value (7.088), suggesting its unfavorable hydrophobicity. In addition, 3z has low gastrointestinal (GI) absorption; this means that it is highly improbable for it to be successfully absorbed from the GI tract into the bloodstream, which can decrease the performance of a drug or limit the nutritional value of a food component. The AMES test (*Salmonella*/microsome) is a widely used assay to assess the potential of a chemical or substance to cause genetic mutations. Therefore, the AMES toxicity refers to the potential of a substance to be mutagenic based on its performance in the AMES test; it should be noted that this does not necessarily mean the substance is toxic in other ways, but it is highlighted for potential long-term health risks, particularly cancer 37. The compound 3z has been tested for its AMES toxicity, which means that it has been evaluated for its ability to induce genetic mutations in bacteria. From Table 4, although the molecule (3z) possesses favorable physicochemical properties, it is evident that it cannot be an orally active drug in humans according to the Lipinski rules. From these findings, it is concluded that molecule 3z does not meet the ADMET criteria. Based on the ADMET results, we modified the tetrahydrobenzo[4,5]thieno[2,3-day]pyrimidine structure by substituting new radicals at the Meta and Ortho positions while removing the ketone functional group to derive new compounds with good ADMET criteria.

**TABLE 4:** ADMET analyses of the drug capivasertib, molecule object (3z), and newly designed molecules.

ADMET/molecules	Rule	M3	M5	Capivasertib	3z
**LogS**	−4 -0.5	−4.00	−4.50	−3.48	−7.84
**LogP**	0–3	2.691	3.54	2.14	7.088
**BBB permeability**		No	No	No	No
**GI absorption**		High	High	High	Low
**D6-sub**		No	No	No	No
**A4-sub**		Yes	No	No	Yes
**A2-inh**		No	No	No	Yes
**C19-inh**		No	No	No	No
**C9-inh**		No	No	No	No
**D6-inh**		No	No	No	No
**A4-inh**		No	No	No	No
**HERG I-inh**		No	No	No	No
**HEGR II-inh**		Yes	No	Yes	No
**Oral rat acute toxicity (LD**_**50**_ **)**		2.061	2.607	2.305	2.725
**Oral rat chronic toxicity (LOAEL)**		1.258	0.759	0.643	−0.795
**Skin sensitization**		No	No	No	No
**AMES toxicity**		No	No	No	Yes

Table 5 represents the six new molecules that were designed to have high biological activity predictions (pIC_50_). Among these molecules, we selected M3 and M5 for further investigations based on the ADMET studies by considering their strongly predicted biological activities. We observed that compound M3 displayed a satisfactory logS value. However, neither of the newly designed molecules were able to penetrate the blood–brain barrier (BBB) effectively. Additionally, both compounds exhibited high GI absorption. The M5 molecule and capivasertib served as substrates for Enzyme A4, while the compound M3 was not used. In addition to functioning as substrates, both M5 and capivasertib act as inhibitors of the HEGR II enzyme. According to the findings presented in Table 5, all compounds comply with the established guidelines for hydrogen-bond acceptor (nHA; ranging from 3 to 5), hydrogen-bond donor (nHD; ranging from 1 to 4), topological surface area (TPSA < 140 Å^2^), and molecular weight (ranging from 428.92 to 514.64 g/mol). Furthermore, the two compounds adhere to Lipinski’s criteria for potential oral bioavailability. In conclusion, the newly designed compounds satisfy the requirements for druglikeness and are promising as potential candidates for the development of novel oral medications.

**TABLE 5:** Physicochemical characteristics and Lipinski’s rule for the new compounds.

P.P	nHA	nHD	TPSA	MW	MR	Synthetic accessibility	Lipinski
Rule	0–12	0–7	0–14	100–600			
M3	5	4	130.39	446.52	133.79	4.66	Yes
M5	5	3	121.60	472.56	143.02	4.85	Yes
Capivasertib	5	4	120.16	428.92	118.97	-	
3z	3	1	102.04	514.64	160.20	-	No

### Design of new molecules targeting the MCF-7 cancer cell line

We utilized 3D-QSAR modeling and conducted ADMET studies to design new compounds that demonstrate improved predicted biological activities. Furthermore, our analysis of the ADMET properties and physicochemical characteristics shows promising results, suggesting that the new compounds possess favorable pharmacokinetic properties (Table 6). Consequently, we believe that the designed molecules have the potential to serve as inhibitors of the MCF-7 cell line and may be considered as candidate drugs.

**TABLE 6:** Design of new inhibitors against the MCF-7 cell line.

Name	Structure	pIC_50_ predicted by 3D-QSAR
**M1**	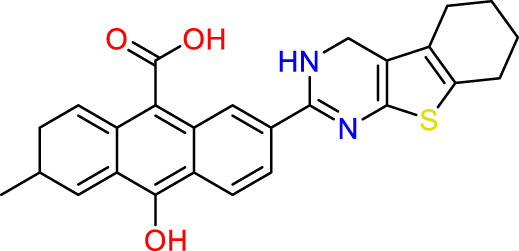	6.21
**M2**	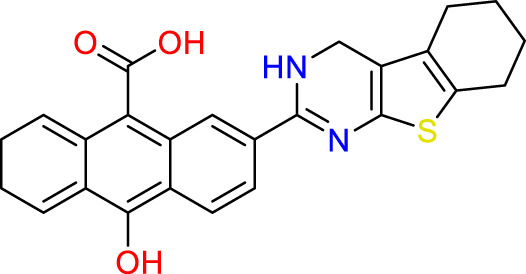	6.14
**M3**	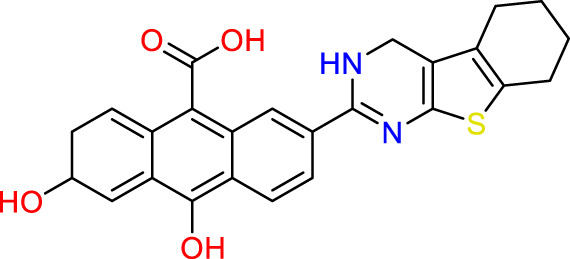	6.21
**M4**	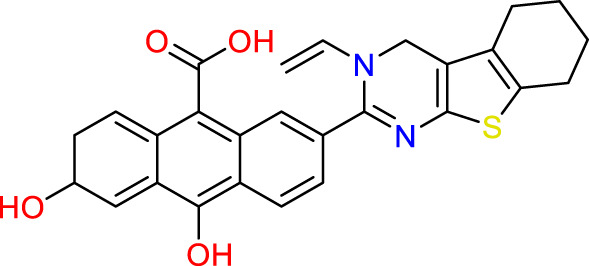	6.18
**M5**	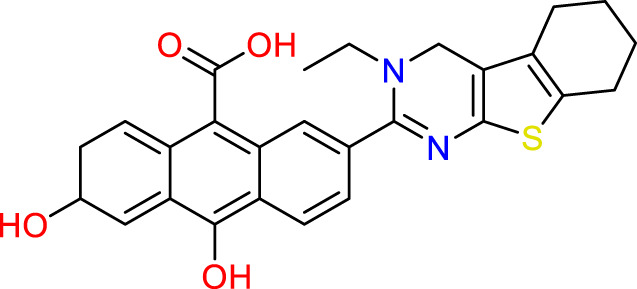	6.18
**M6**	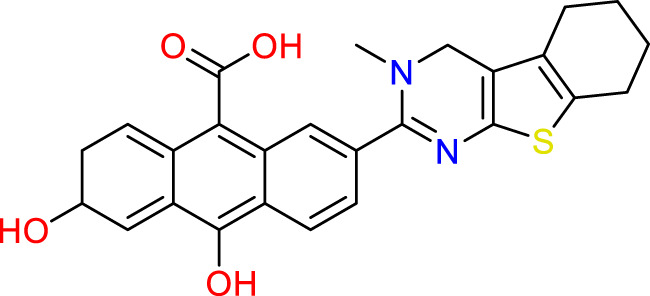	6.18

### Molecular docking studies

**FIGURE 6:**
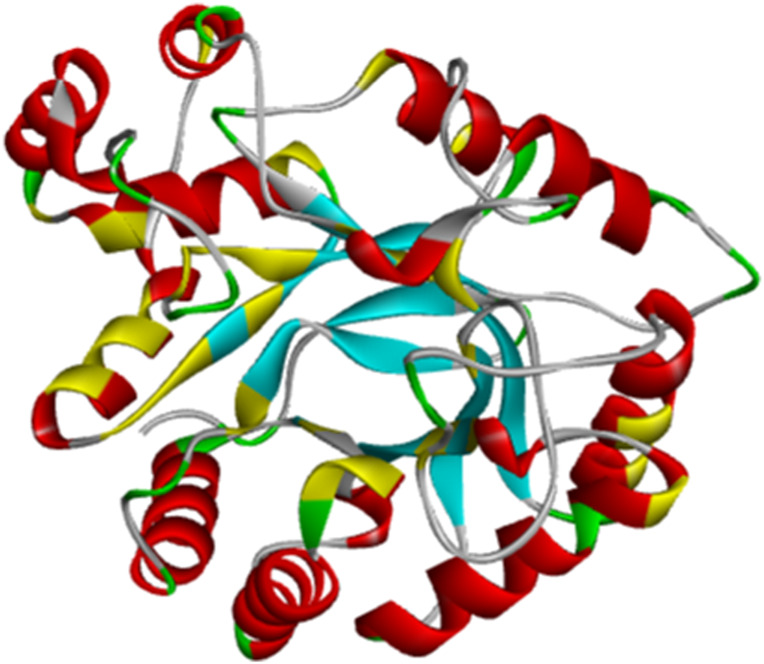
Topology of the ERα protein (PDB ID: 4XO6, chain A).

**FIGURE 7:**
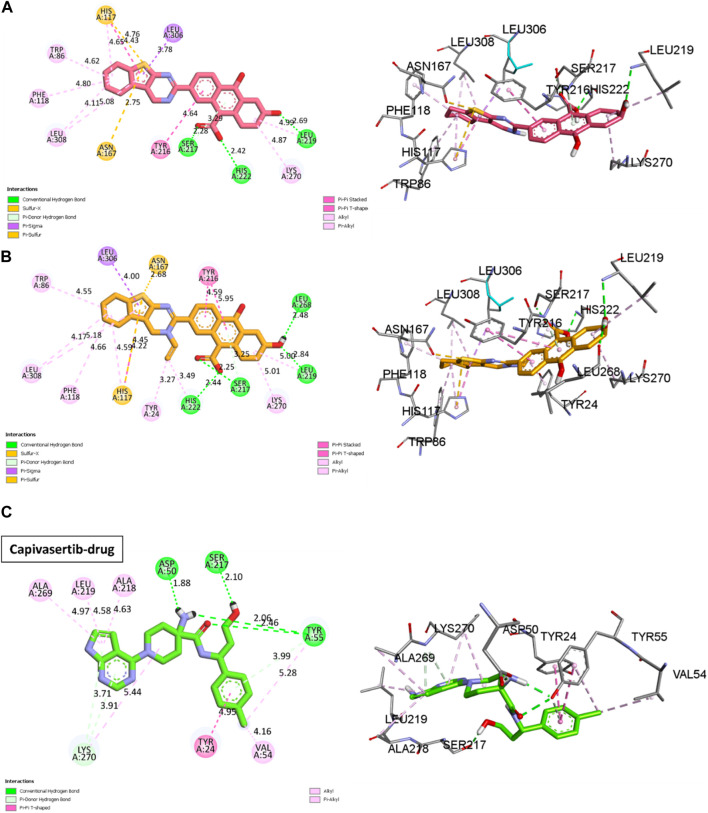
2D and 3D visualizations of the docking results for the newly designed compounds **(A)** M3 and **(B)** M5 against the MCF-7 breast cancer cell line. **(C)** Reference 2D and 3D visualizations of the docking results for the FDA-approved drug capivasertib.

Molecular docking is based on algorithms that anticipate the most stable conformation of the ligand–protein complex depending on the binding energy. We performed molecular docking studies for the most active compounds and capivasertib, which is an FDA-approved drug, to compare the docking interactions and performances between the new compounds with the receptor and drug capivasertib having the same receptor. We performed docking simulations to examine the interactions between our predicted molecules and the protein ERα (PDB ID: 4XO6) Figure 6. The findings of the simulations for the two selected compounds (M3 and M5) and capivasertib are shown in Figure 7A–C, respectively. The initial observations visually demonstrate that M3 has three hydrogen bonds involving residues Leu219, His222, and Ser217; this is attributed to the polarity of OH- and presence of a lone electron pair on the oxygen, which act as electron acceptors during formation of hydrogen bonds. However, M5 exhibits a distinct feature wherein it forms an extra hydrogen bond with residue Leu268; moreover, it engages in alkyl and π-alkyl interactions with Trp86, Leu308, Peh118, and Lys270. These interactions occur when the alkyl group of the compound interacts with a pi system within the receptor due to the hydrophobic nature of the cyclohexane and cyclopenta-1, 3-diene moieties. The presence of the penta-1, 3-diene aromatic group in the ligands (M3 and M5 molecules) leads to π-sigma interactions with a positively charged residue (Leu306), which affects the stability of the ligand–molecule complex. These interactions are significant as they can greatly influence the binding affinities and specificities of the ligands.

Other interactions are also noted, like the π-sulfur case between the thiophene and residues Asn167 and His117; these interactions occur when the delocalized π-orbitals of the thiophene ring interact with the lone electron pairs of the sulfur atom in the π-sulfur ligand. This may contribute to the affinity of the bond in the docking results by stabilizing the complex through orbital riding and electron delocalization.

The docking results of capivasertib shown in Figure 7C and reveal four hydrogen bonds involving specific residues and functional groups. One hydrogen bond is formed between the residue Ser50 and OH functional group of capivasertib. Another hydrogen bond is formed between the residue Ser217 and Cl atom of capivasertib. Additionally, two hydrogen bonds are observed with the Tyr55 residue, one involving the OH functional group and the other involving the ketone functional group of capivasertib. These hydrogen bonds play crucial roles in the interactions between capivasertib and its target molecules. We observed that the interaction patterns between our newly designed drugs (M3/M5) and capivasertib are identical. Specifically, we note the presence of two interactions: a hydrogen bond with Ser217 and a π-π interaction involving Tyr24. By comparing the results obtained from M3 and M5 with the docking results of capivasertib, we deduce that both M5 and capivasertib interact with two similar residues, namely, Ser217 and Tyr24. On the other hand, M3 only interacts with one residue, which is Ser217. This suggests that the compounds have the potential to bind effectively with the target biomolecules. According to literature, the residues Leu219, His222, and Ser217 also play very important roles in studies on anticancer activities (Zouaghi et al., 2023). This is a positive sign for drug development because successful binding can influence the desired therapeutic effect.

### MD simulation

MD simulations were conducted to comprehensively investigate the stabilities of the newly designed molecules in a complex with the protein. Such simulations have been used in many studies to explain atomic-level variations within the protein–ligand complex, capturing the dynamic interactions between the molecules in a dynamic medium (Kumari et al., 2022). In the following study, we examined several parameters such as the RMSD, RMSF, RoG, SASA, and hydrogen-bond formation for the M3 and M5 complexes during 100 ns of molecular simulation.

**FIGURE 8:**
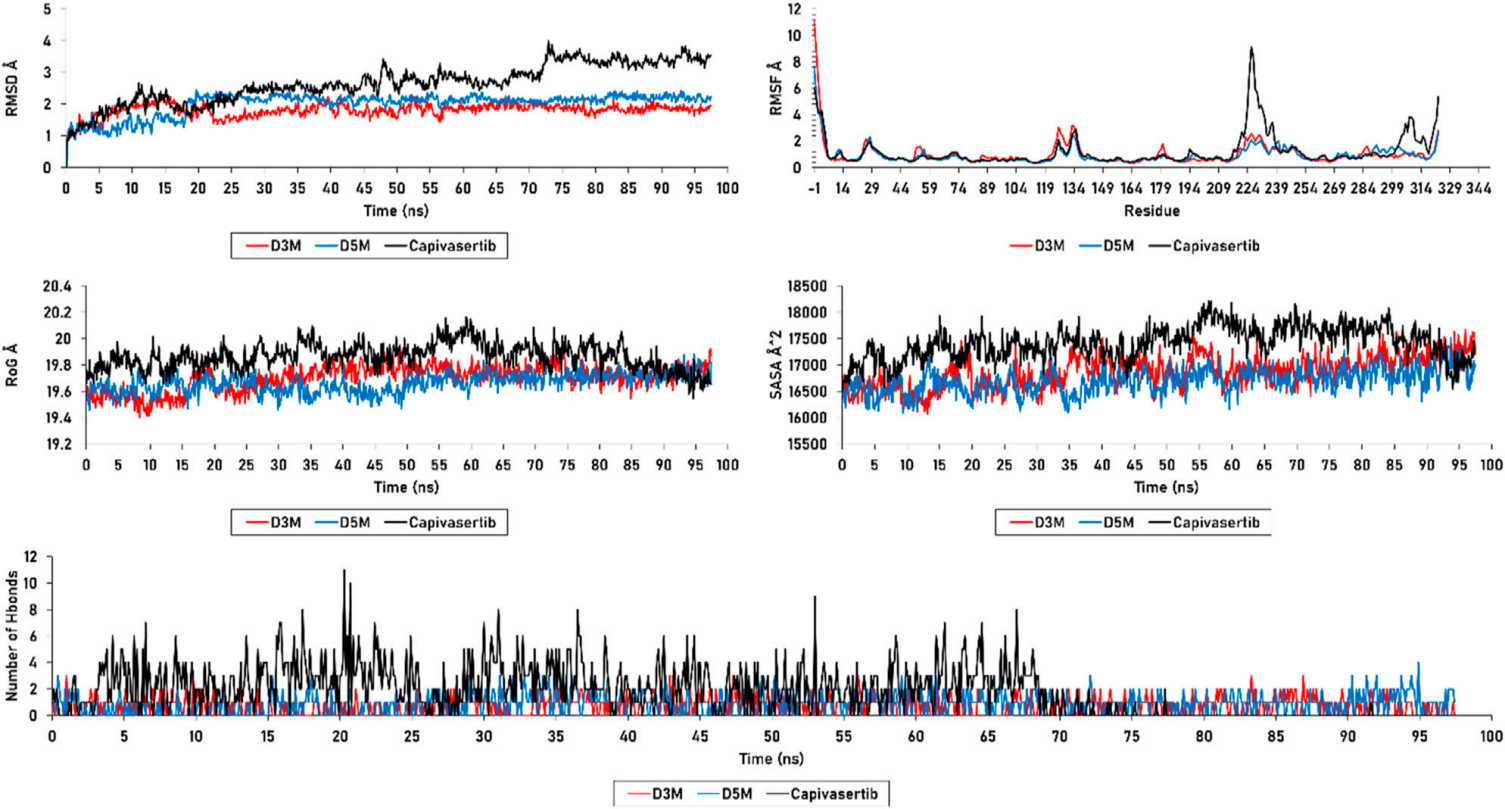
RMSD, RMSF, SASA, RoG, and H-bonds of the D3M and D5M complexes, as well as capivasertib.

The results achieved for the RMSD, RMSF, RoG, SASA, and H-bonds are presented in Figure 8. The RMSD investigation for the D3M (M3–ERα) and D5M (M5–ERα) complexes show average distances between 1 and 2.3 Å. Both complexes vary from 1 to 2.3 Å and thereafter stabilize at 2 Å during the first 20 ns. The RMSD analysis demonstrates that D3M and D5M are more effective in promoting stability than capivasertib. However, some fluctuations are observed in the RMSD plot, such as a fluctuation in the D3M complex from 1.5 to 1.2 Å at approximately 40 ns. The D5M complex demonstrates strong stability over a period of 60 ns, with low fluctuations from 1 to 2.3 Å in the first 40 ns, after which it remains stable at 2 Å. In contrast, the reference molecule capivasertib experiences fluctuations from 1 to 2.8 Å over a period of 40 ns.

Furthermore, the complex capivasertib–ERα exhibits an increase in fluctuation from 2.7 Å to 3.8 Å between 45 ns and 75 ns. Good stabilities of the D3M and D5M complexes are observed from 80 ns to 100 ns at 1.8 Å, while the reference molecule capivasertib experiences a range of fluctuations from 1 to 3.8 Å over a period of 100 ns. The RMSD plots show that the compounds M3 and M5 exhibit good stabilities with the ERα protein compared to capivasertib.

The RMSF values were calculated to assess the flexibility of the protein structure after adjustment to the reference frame. Higher RMSF values suggest that the protein’s loops and turns are poorly structured, whereas lower flexibility indicates the presence of more stable and organized regions. The RMSF analysis revealed similar stabilities for the residues of the complexes formed by the new compounds with ERα. Table 7 presents the residues with RMSF values less than 3 Å that contribute to the stability of the newly designed molecules. In particular, the Glu224 residue in capivasertib exhibits an RMSF value of 4.557291, followed by Glu227 with an RMSF value of 8.392049. These values explain the peaks observed in Figure 8, which are located outside the active sites of ERα compared with D3M and D5M.

**TABLE 7:** Residues with RMSF values less than 3 Å.

N°	Residue	D3M	D5M	Capivasertib
14	ASP	0.565390	0.727055	0.781049
29	SER	1.496763	1.682218	1.531807
44	TYR	0.700645	0.682867	0.678045
59	GLN	0.932650	0.799256	0.681637
74	VAL	0.801856	0.731317	0.909452
98	LEU	0.738380373	0.532187521	0.5297966
104	ASN	0.791040	0.613014	0.666166
119	HIS	0.578250	0.477338	0.595151
134	PHE	3.117864	2.372437	2.705154
149	PRO	0.519812	0.525314	0.602962
179	PHE	1.326364	0.730985	0.893380
209	ALA	0.644678	0.767425	0.736610
224	GLU	2.018936	1.457529	>3
239	PRO	1.502835	1.847240	1.512882
254	LYS	0.813202	0.625272	0.764170
269	LYS	0.524341	0.672288	0.597879
284	AGLU	1.189149	0.878250	0.889363
299	ALA	0.910628	1.563716	1.008637
314	LYS	1.055927	0.734300	1.826706

The plots of SASA and RoG exhibit that the compounds M3 and M5 maintain their structure stabilities during the 100 ns simulation; both molecules are observed to be stable on the SASA plot, with an average value of 16,800 Å^2^. This pattern is also observed in the RoG calculation, which yields an average value of 19.8 Å. However, in the case of capivasertib, the SASA calculation shows an average value of 18,300 Å^2^ with noticeable fluctuations; this remarkable variation significantly affects both the molecular surface of capivasertib and its interactions with the solvent. However, the RoG analysis reveals a fluctuation of 20 Å initially, followed by stabilization at 19 Å; this suggests that capivasertib is unable to maintain its compactness throughout the simulation. The data presented in Figure 8 demonstrate the favorable stabilities of the n-hydrogen bonds of the newly designed molecules as they do not exceed four hydrogen bonds over 100 ns. Importantly, these bonds do not show any unnecessary or additional occupancies. However, capivasertib exhibits significant variation, particularly between 15 ns and 25 ns, where the count of hydrogen bonds reaches ten, allowing additional occupancies. Additional variations are observed between 30 ns and 40 ns and between 60 ns and 70 ns; these observations are further supported by the results of the RMSD, RMSF, SASA, and RoG analyses.

The MD simulation results of the RMSD, RMSF, SASA, and RoG calculations substantiate the strong affinities of the newly designed models towards ERα. Additionally, these simulations confirm the remarkable stabilities of the models, as evidenced by their consistent behaviors throughout the 100 ns of simulation. Essential dynamics analysis was performed to examine the significant movements that occur after a ligand is bound to the protein. By comparing the images at 0 ns, 50 ns, and 100 ns, the dynamic differences among M3, M5, and capivasertib can be visualized easily.

**FIGURE 9:**
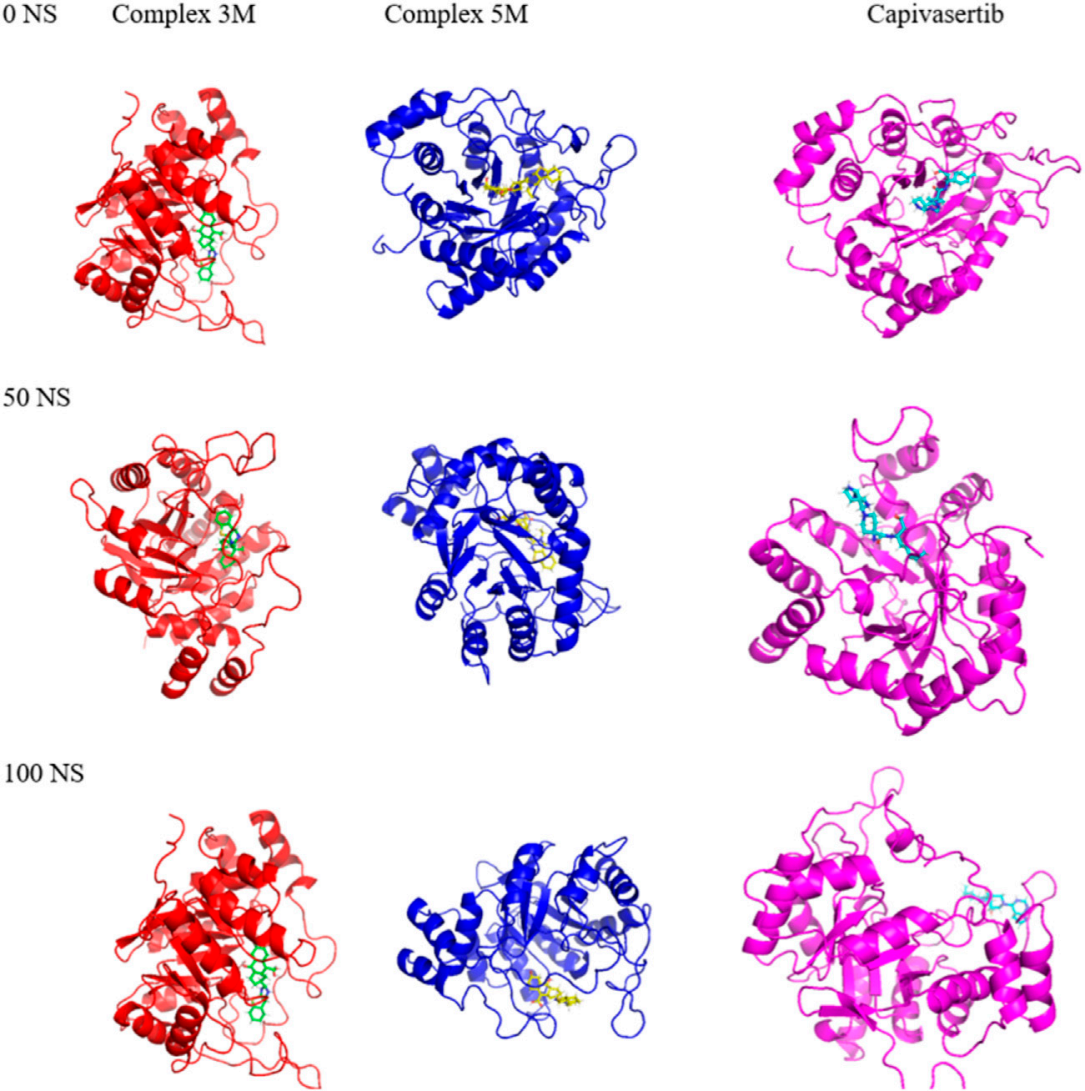
Mappings of the conformations of the M3, M5, and capivasertib complexes at 0 ns, 50 ns, and 100 ns from the MD simulations.

Based on the 2D projection map in Figure 9, it is clear that inhibitor binding leads to formation of stable complexes. Among the complexes, M3 is the most stable, as evidenced by its highly stable cluster and minimal occupation of the phase space compared with the other molecules. In contrast, capivasertib has the lowest stability out of all the compounds identified. This observation is further confirmed by the representative illustrations.

### Free binding energy (MM/GBSA)

We input snapshots from the MD results obtained during a 100-ns trajectory to the MM-GBSA method. From Table 8, it is evident that ΔE_EL_ for capivasertib is larger than the values of the new compounds. This leads us to the conclusion that capivasertib is relatively less stable compared to D3M and D5M. The newly designed compounds exhibit higher values of van der Waals interactions than capivasertib. This indicates that the strengths of the van der Waals interactions in D3M and D5M are larger. The negative energy surface interaction, ΔE_SUR_, typically indicates an attractive force. The D3M complex exhibits favorable energy surface interaction; the greater the negative energy of a compound in the gas phase, the better its gas-phase power. All compounds demonstrate favorable energies in the gas phase. Specifically, capivasertib exhibits a strong gas-phase interaction owing to its ΔE_SURF_ value. Positive values of energy solvation, ΔE_Sol_, enhance the stabilities of the compounds; the new compounds exhibit positive energies when combined with the complexes D3M and D5M, unlike capivasertib that has a lower ΔE_Sol_ value. This indicates that the new compounds possess lower solvation energies. The ΔE_GB_ results show that the powers of all compounds are involved in cavitation/solvation; here, D5M exhibits a lower ΔE_GB_ than D3M and capivasertib, which implies that D5M may have a decreased ability to solvate that in turn enhances its binding efficiency. Although capivasertib’s total energy is positive, both D3M and D5M exhibit negative total energies. This suggests that D3M and D5M have more advantageous binding affinities than capivasertib, indicating their superior efficacies as ERα inhibitors in MCF-7 cells. The MM/GBSA results suggest that the newly designed compounds M3 and M5 show more beneficial interactions with the target protein than capivasertib. Molecule M3 exhibits highly promising outcomes, as evidenced by its favorable total energy and superior binding energy in comparison to the other compounds.

**TABLE 8:** Free energy binding values of the complexes based on the MM/GBSA method.

ΔE (kcal∙mol^-1^)	DM3	DM5	Capivasertib
ΔE_GB_	291.88	129.60	393.17
ΔE_SURF_	−7.34	−5.93	−4.44
ΔE_EL_	−294.01	−98.85	347.08
ΔE_Gas_	−337.84	−141.88	−367.25
ΔE_Sol_	284.53	123.66	388.73
Δ_VWAALS_	−43.83	−43.02	−20.17
ΔE_Total_	−53.31	−18.21	21.48

## Conclusion

In11 this study, new potential drugs were investigated using molecular modeling techniques against breast cancer, which is one of the most prevalent diseases worldwide. The primary aim of the study was to identify MCF-7 inhibitors, which are crucial cell lines in the treatment of breast cancer. The 3D-QSAR method was employed to predict new MCF-7 inhibitors, resulting in the generation of two strong models with good results in identifying groups that may interact with each other. The two established models were evaluated successfully using external validation methods. Analysis of the various contour maps of both models allowed us to identify the functional groups that could enhance the inhibitory abilities. We utilized ADMET studies and pharmacokinetics characteristics to design six inhibitors based on their CoMFA and CoMSIA predictions, with the addition of OH functional groups. The presence and position of the OH group can affect the binding affinity, selectivity, and efficacy of these molecules at the target sites, allowing strong intermolecular interactions with other polar molecules. Hydrogen bonding is critical for many biological processes such as protein folding. Out of the six inhibitors developed, two molecules exhibited good interactions with the target protein in molecular docking simulations. To validate these findings, the data were subjected to MD simulations and binding free energy calculations based on the MM/GBSA over a duration of 100 ns. In conclusion, this research provides important insights into the development of novel drugs to combat breast cancer. The results of this study are expected to be of foundational value for further research and development of inhibitors as promising drugs for breast cancer treatment.

## Data Availability

The original contributions presented in the study are included in the article/supplementary material; further inquiries can be directed to the corresponding author.
